# Embolic Protection Devices in Saphenous Vein Graft and Native Vessel Percutaneous Intervention: A Review

**DOI:** 10.2174/157340312803217201

**Published:** 2012-08

**Authors:** Eron Sturm, David Goldberg, Sheldon Goldberg

**Affiliations:** Department of Cardiovascular Medicine, Hahnemann University Hospital, Philadelphia, Pennsylvania

**Keywords:** Embolic protection devices, microvascular obstruction, no-reflow, plaque embolization, saphenous vein grafts, thrombus embolization.

## Abstract

The clinical benefit of percutaneous intervention (PCI) depends on both angiographic success at the site of intervention as well as the restoration of adequate microvascular perfusion. Saphenous vein graft intervention is commonly associated with evidence of distal plaque embolization, which is correlated with worse clinical outcomes. Despite successful epicardial intervention in the acute MI patient treated with primary PCI, distal tissue perfusion may still be absent in up to 25% of cases [1-3]. Multiple devices and pharmacologic regimens have been developed and refined in an attempt to protect the microvascular circulation during both saphenous vein graft intervention and primary PCI in the acute MI setting. We will review the evidence for various techniques for embolic protection of the distal myocardium during saphenous vein graft PCI and primary PCI in the native vessel.

## THROMBUS FORMATION AND ADVERSE CONSEQUENCES OF DISTAL EMBOLIZATION 

The most frequent cause of ischemic heart disease is coronary atherosclerosis, which manifests in the acute presentation as plaque rupture with superimposed thrombus. The thrombi in acute coronary syndrome (ACS) and ST-segment elevation myocardial infarction (STEMI) are characterized by densely packed fibrin; in STEMI, the majority of the occlusive thrombus is made up of a loose network of fibrin and red cells, with a platelet component observed in the setting of sudden plaque rupture. The non-occlusive thrombus in ACS is more likely to be a mural thrombus covered by platelets [4]. On angiography, the presence of thrombus is defined as a filling defect with either a total occlusion or partial occlusion with convex, irregular, or hazy-appearing margins, which can also exhibit contrast retention or staining at the site of the filling defect [5]. 

The presence of angiographically detectable thrombus is associated with a higher incidence of in-hospital and long-term major adverse cardiac events [6-9]. Coronary thrombus may cause myocardial injury either via spontaneous or PCI-induced distal embolization, resulting in myocardial infarction. In the cardiac catheterization laboratory, impaired microvascular perfusion is manifest as the “no-reflow” phenomenon. Even after successful PCI, tissue perfusion at the level of the myocardium is still not restored in approximately one-third of cases, due to impairment in microvascular blood flow [10-13]. Patients who develop distal embolization have lower procedural success rates, larger infarcts, and most importantly, increased in-hospital and late mortality [11, 14]. The size of the angiographic thrombus is also associated with a negative outcome; large thrombus burden is an independent predictor of distal embolization, resulting in lower TIMI flow rates, worse myocardial blush grades, and higher 2-year mortality and adverse event rates [7, 14]. Angiographic indicators of embolization such as corrected Thromboloysis in Myocardial Infarction (TIMI) frame count, myocardial blush grade, and the completeness of ST-segment resolution are highly predictive of clinical outcomes [15-16]. 

## EMBOLIC PROTECTION DEVICES IN SAPHENOUS VEIN GRAFT PERCUTANEOUS INTERVENTION 

**Fig. (1):**
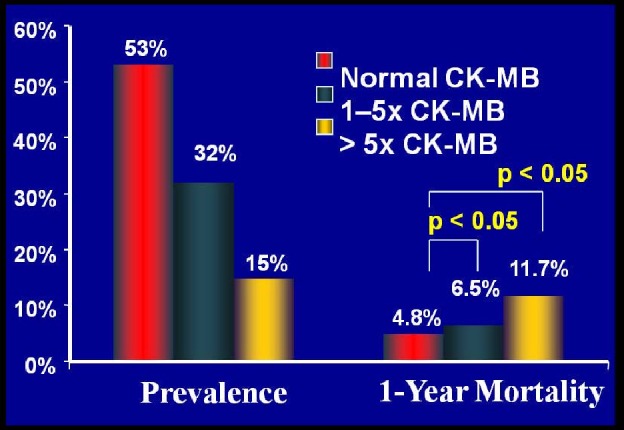
The benefits of prevention of microvascular obstruction with embolic protection devices in saphenous vein graft intervention is illustrated in this figure. Note that the degree ofelevation in CK-MB level is associated with increased 1-year mortality [17].

**Fig. (2):**
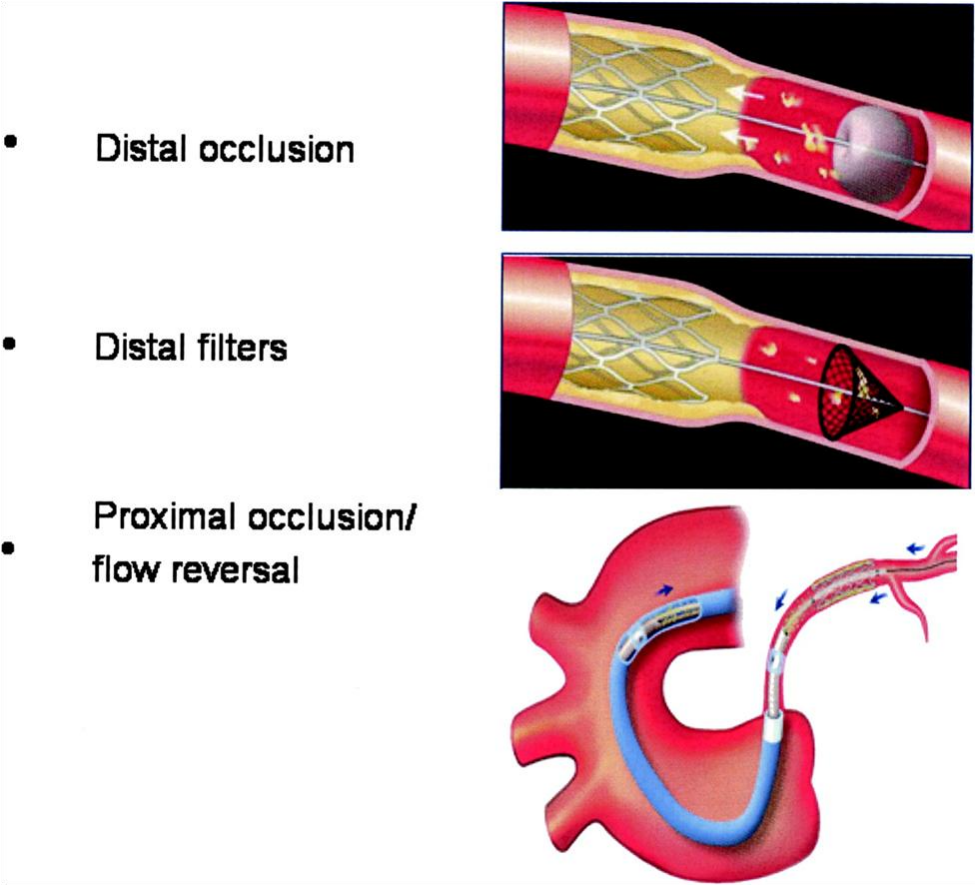
Diagrams depicting the three primary types of embolic protection devices (EPDs) currently utilized for saphenous vein graft intervention and native vessel percutaneous intervention for primary PCI. Top: distal occlusion balloon with aspiration; middle: distal filter protection; bottom: proximal balloon occlusion with aspiration. Reprinted with permission [24].

Atherosclerotic plaques in saphenous vein grafts are more diffuse, friable, and contain more inflammatory cells, have absent or small fibrous caps, and little or no calcification relative to native coronary atherosclerotic vessels. These characteristics predispose SVGs to extensive thrombotic burden and distal embolization during percutaneous intervention, resulting in the no-reflow phenomenon and distal microvascular obstruction. In an early study of SVG PCI prior to the routine use of distal protection, the degree of CK elevation was strongly associated with one-year mortality (Fig. **1**) [17]*. *Accordingly, trials of myocardial protection with Embolic Protection Devices (EPD) were originally used in the setting of saphenous vein graft (SVG) PCI (Table **1**). These devices have subsequently been evaluated in the setting of native vessel primary PCI. The pivotal trial demonstrating the efficacy of embolic protection was the Saphenous vein graft Angioplasty Free of Emboli Randomized (SAFER) trial, which used a distal occlusion balloon and a separate aspiration catheter to retrieve debris liberated during PCI[18]. Currently, there are three primary types of EPDs: distal balloon occlusion and aspiration system; distal filters; and proximal balloon occlusion and aspiration system (Fig. **2**)*. *

### Distal Occlusion/Aspiration System

**Fig. (3):**
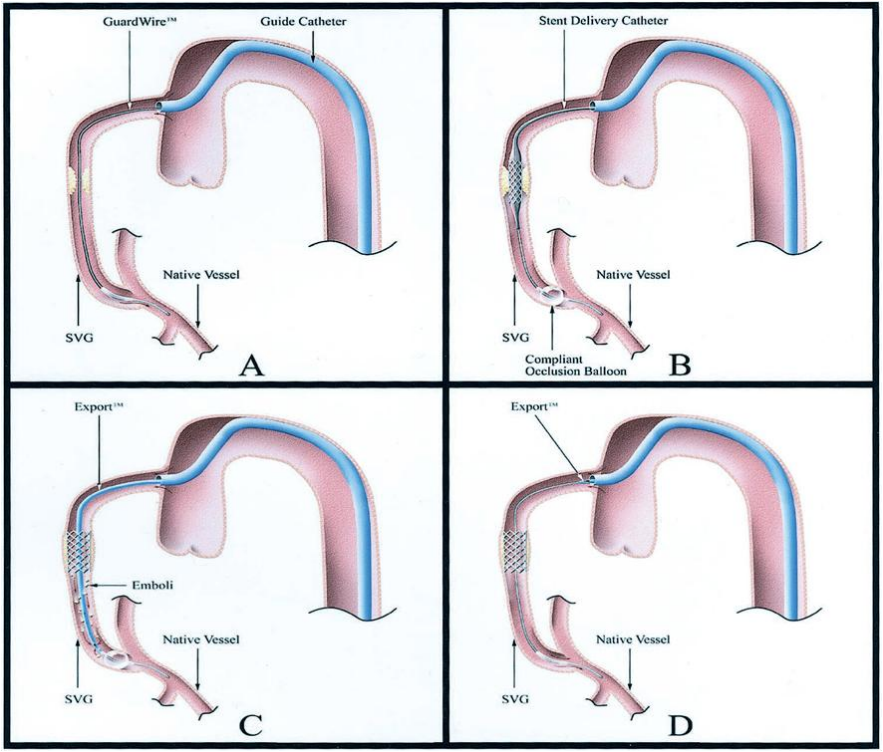
Diagram of the GuardWire distal occlusion/aspiration system. (**A**) The GuardWire is advanced from the guide catheter, through and beyond the saphenous vein graft (SVG) lesion. (**B**) The compliant occlusion balloon at the GuardWire tip is inflated to occlude flow before the stent is deployed. (**C**) After stent deployment, an Export catheter is advanced over the GuardWire and aspirated to remove stagnant column of blood with suspended embolic debris. (**D**) The GuardWire balloon is deflated to restore antegrade blood flow. Diagram and description from the SAFER trial [18].

The PercuSurge GuardWire system occludes the target vessel several centimeters distal to the target lesion during SVG PCI in order to provide myocardial protection. After stent placement, aspiration removes debris-laden blood prior to balloon deflation and restoration of antegrade blood flow (Fig. **3**). The PercuSurge system was the first EPD to gain Food and Drug Administration (FDA) approval following the results of the Saphenous Vein Graft Angioplasty Free of Emboli Randomized (SAFER) trial [23]. This landmark study showed an impressive 42% reduction in 30-day MACE as well as a marked decrease in the incidence of the no-reflow phenomenon. Following the SAFER trial, the TriActiv system was approved by the FDA after proving its noninferiority in the Protection During Saphenous Vein Graft Intervention to Prevent Distal Embolization (PRIDE) trial [21]. 

### Distal Filtration System

The FilterWire EX Randomized Evaluation (FIRE) study showed noninferiority of the FilterWire EX System to the GuardWire balloon occlusion and aspiration system, which led to FDA approval of the first distal filtration device [20]. Subsequent investigation with newer generation devices showed similar results. The Embolic Protection Transluminally with the FilterWire EZ Device in Saphenous Vein Grafts (BLAZE I and II) study showed a decrease in MACE with a second generation of the FilterWire EX [25] and the Saphenous Vein Graft protection In a Distal Embolic Protection Randomized Trial (SPIDER) study showed noninferiority of the Spider Rx filtration device compared to GuardWire and FilterWire [26]. The Assessment of the Medtronic AVE Interceptor Saphenous Vein Graft Filter System (AMEthyst) trial examined another filter, the Interceptor PLUS, which was also shown to be noninferior to the GuardWire and FilterWire EZ [22]. 

### Proximal Occlusion/Aspiration System

**Fig. (4):**
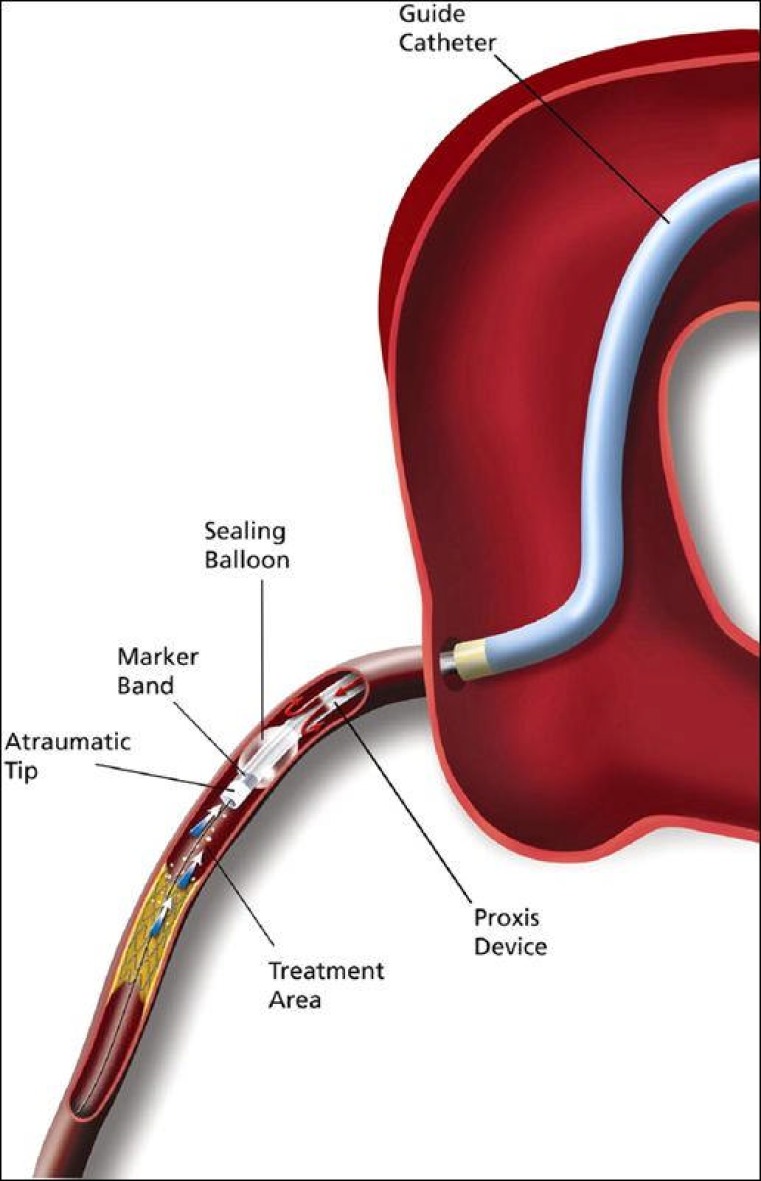
The Proxis Embolic Protection System. The Proxis device is tracked through the guide catheter and into the target vessel, proximal to the treatment area. The guidewire and interventional device are inserted through Proxis and may be staged proximal to the treatment area before balloon inflation. Balloon inflation suspends blood flow, ensuring stagnation of blood and liberated embolic material during treatment of the lesion. During protection, a static column of contrast verifies adequate sealing and highlights the treatment area to facilitate interventional device placement (diagram and description from the PROXIMAL trial) [23].

Proximal occlusion devices occlude the vessel proximal to a target lesion and suspend antegrade flow. Following intervention, the stagnant blood and debris are then aspirated. The only FDA approved proximal occlusion/aspiration device is the Proxis system (Fig. **4**). This approval was based on the PROXIMAL trial that showed the Proxis system to be noninferior to distal EPD [23]. Patients with stenosis in the distal portion of a saphenous vein graft, without an adequate landing zone for a distal protection device, had been previously ineligible for embolic protection. Since the Proxis system does not require a distal landing zone, it is suited for patients with distal saphenous vein graft lesions, and expands the number of cases of SVG PCI that are conducive to embolic protection by nearly 20% [27]. 

In light of the evidence, the American College of Cardiology guidelines give a class I recommendation for the use of EPD in SVG PCI when technically feasible [28]. Despite the guidelines, registry data show that EPDs are utilized in only 22% of SVG PCI [29]. This may be due in part to anatomic difficulties, such as vessel tortuosity, vessel diameter, and the absence of an adequate non-diseased landing zone. Additionally, the important benefit of filter devices over occlusion balloon systems is the preservation of antegrade flow. In cases of severe left ventricular dysfunction or when large areas of myocardium are subtended by the target vessel, even brief period of ischemia with an occlusion balloon system may not be tolerated. Following the proof of concept with the use of EPDs in diseased SVGs, it was intuitive to attempt to apply these devices to protect the myocardium during PCI of highly thrombotic native vessel coronary lesions. 

## EMBOLIC PROTECTION DEVICES IN NATIVE VESSEL PERCUTANEOUS INTERVENTION 

PCI has been shown to be the preferred strategy over fibrinolysis in STEMI, reducing the combined outcome of in-hospital re-infarction and death [30-31]. Distal micro and macro-embolization may limit the effectiveness of myocardial reperfusion and lead to larger infarct size and increased mortality [10-11]. As a result, interventional devices were developed in an attempt to reduce emboli using two methods: distal embolic protection devices designed to capture thrombotic debris and thrombectomy devices aimed at direct thrombus extraction. 

The Enhanced Myocardial and Efficacy and Recovery by Aspiration of Liberated Debris (EMERALD) trial was the pivotal trial of EPD in patients with STEMI presenting within 6 hours of symptoms. A total of 501 patients were randomized to receive PCI with the GuardWire versus conventional PCI. Although the device reduced the incidence of angiographic slow/no-reflow, there was no significant effect on other surrogate or hard clinical endpoints: ST-segment resolution at 30 minutes, infarct size, as well as MACE rates were all similar between the study and the control group [32]. The randomized PCI Treatment of Myocardial Infarction for Salvage of Endangered Myocardium (PROMISE) trial randomized 200 acute MI patients (both STEMI and non-ST elevation MI patients) with evidence of angiographic thrombus to distal protection with the FilterWire-EX. There was no difference in the primary endpoint of maximal adenosine-induced Doppler flow velocity in the infarct related artery after recanalization. There was also no difference in infarct size, as measured by delayed enhancement on nuclear MRI, or on 30-day mortality versus PCI alone [33]. A third major randomized trial of distal protection, the Drug Elution and Distal Protection in ST-Elevation Myocardial Infarction (DEDICATION) trial, was a randomized comparison of 626 patients with STEMI using the FilterWire or the SpiderX filter devices in native vessels versus primary PCI. Similar to prior studies, this trial showed no difference between the EPD group and the conventional PCI group in terms of left ventricular wall motion index, ST-segment resolution, or cardiac biomarkers [34]. In the PREPARE (Proximal Embolic Protection in Acute MI and Resolution of ST-Elevation) trial, the use of the Proxis proximal protection device was compared to conventional PCI and showed improved microvascular flow as reflected by improved ST-segment resolution in the proximal protection arm [35]. Results from smaller randomized trials and registry data using various EPDs in native vessel PCI have shown equally disappointing clinical results (Table **2**) [33, 36-37]. 

Several mechanisms have been proposed for the negative results of EPDs in native coronary vessel thrombotic lesions: the bulky nature of these EPD devices may themselves cause embolization, the presence of side branches that cannot be protected, incomplete protection with the device due to either incomplete device/vessel apposition or pore size that fails to capture particles <100μm within the filter, and predilation is often necessary to facilitate delivery of the device, limiting the benefit as distal embolization occurs during the pre-dilation balloon inflation [13, 37]. In addition, the EPD might only be beneficial in patients with large thrombus burden without multiple side branches proximal to the protection device. 

## DIRECT THROMBECTOMY TO PROTECT THE DISTAL MYOCARDIUM IN NATIVE VESSEL PERCUTANEOUS INTERVENTION 

As an alternative to embolic protection devices, devices aimed at direct thrombus extraction were designed to capture thrombotic debris in native vessel PCI and protect the distal myocardium. Direct thrombectomy devices are classified based primarily on their mechanism of action. The two primary types are either manual or mechanical aspiration. All of the thrombectomy devices have shown benefit in terms of surrogate end-points such as angiographic TIMI flow, infarct size reduction, ST-segment resolution, and biomarker analysis [14, 42-49]. Currently, however, only manual aspiration has compelling data demonstrating clinical benefit in the STEMI population [50-51]. 

Manual aspiration catheters are appealing as they are quick and easy to use, as well as relatively inexpensive. Of the thrombectomy devices, the manual aspiration catheter is the only one to show a clinical benefit for patients in the STEMI setting. The first randomized trial was REMEDIA, which examined manual thrombectomy as an adjunct to primary PCI, and showed improvement in the surrogate endpoints of myocardial blush grade, ST-segment resolution, and decreased incidence of slow and no-reflow [42]. The Thrombus Aspiration during Primary Percutaneous Coronary Intervention in Acute Myocardial Infarction Study (TAPAS) was a landmark randomized trial comparing conventional PCI to thrombus aspiration as the initial step plus conventional PCI in the STEMI population. The primary outcome was a myocardial blush score of 0 or 1, which occurred in 17.1% of the patients in the thrombus-aspiration arm and in 26.3% of those in the conventional PCI arm (P<0.001). Thrombus aspiration prior to PCI also improved the frequency of complete ST-segment resolution in the thrombus-aspiration arm (56.6% versus 44.2% in the conventional PCI arm, P<0.001). Importantly, patient follow-up at 30 days revealed improved patient outcomes that correlated to the improved surrogate endpoints. Improved myocardial blush scores seen in the thrombus-aspiration group correlated to lower death rates. The mortality rates were 5.2%, 2.9%, and 1.0% for myocardial blush grade of 0 or 1, 2, and 3 respectively (P=0.003). In addition, adverse events occurred in 14.1%, 8.8%, and 4.2% for myocardial blush grade of 0 or 1, 2, and 3 respectively (P<0.001) [3, 52]. 

Although the landmark TAPAS trial of manual aspiration used surrogate endpoints to assess the outcomes of thrombus-aspiration prior to PCI in the STEMI patients, the surrogate endpoints were clearly associated with rates of death and MACE events, which supports the validity of using such endpoints in this STEMI population. Manual thrombectomy ultimately resulted in an improvement in surrogate markers of myocardial reperfusion and clinical outcomes in the patient who presents with an ST-elevation myocardial infarction as compared to conventional PCI [3]. Multiple smaller trials of manual thrombectomy with different devices utilizing the manual aspiration technique (EXPIRA, DEAR-MI, and PIHRATE) showed similar improvements in surrogate endpoints and a trend towards clinical benefit in the STEMI population [43, 47, 48, 52-54]. 

The Angiojet rheolytic thrombectomy (RT) system is the primary mechanical aspiration device in use. RT uses high-velocity saline jets around the catheter tip to entrain thrombus towards the inflow windows, fragmenting the thrombus and evacuating it from the vessel. Multiple randomized trials comparing mechanical thrombectomy as an adjunct to PCI versus PCI alone showed no significant benefit for mechanical thrombectomy [55-56]. However, a single center study of RT in the subset of STEMI patients with large (Grade 4) thrombus burden, found an improvement in MACE-free survival with mechanical thrombectomy compared to conventional PCI without RT [7, 57]. Although mechanical thrombectomy has not shown a clinical benefit in all comers, this may identify a subset of STEMI patients with a large thrombus burden that may benefit from mechanical thrombectomy. A large meta-analysis of 17 randomized trials of direct thrombectomy (both mechanical and manual) as an adjunct to PCI versus conventional PCI, showed that there was no difference in mortality between the two strategies. A subgroup analysis of this population showed a trend toward higher mortality with the use of mechanical devices, while manual aspiration showed a significant trend toward reduction in mortality [51]. 

## CONCLUSIONS 

Both saphenous vein graft intervention as well as primary PCI of the thrombotic native coronary artery in the acute MI setting are associated with important risk of distal embolization, which limit the clinical benefit to the patient. Various techniques have been developed to protect the microvascular circulation during these interventions using embolic protection devices and thrombectomy devices, as well as pharmacologic methods to prevent and reverse no-slow reflow phenomenon. In SVG intervention, distal protection with a balloon-occlusion/aspiration systems or a filter-based device have proven clinical benefits and should be used when technically feasible. The evidence for distal protection in the native coronary artery shows some improvement in angiographic results. However, this does not translate into a clinical benefit and thus, is not routinely recommended at this time. 

Emerging strategies targeting reperfusion injury at the tissue level, which activate intracellular cardioprotective mechanisms to improve tissue perfusion and improve outcomes, may be an adjunct to care for the patient with distal embolic phenomenon [58]. Novel intracoronary imaging techniques to detect those “lipid-rich” plaques [59] that may be pre-disposed to distal embolization may allow us to tailor future treatment for those patients at highest risk of distal embolization. 

## Figures and Tables

**Table 1.** Randomized Trials of Embolic Protection Devices in Saphenous Vein Graft Intervention

Trial/Reference	n	Device	GP IIb/IIIa use	Primary Endpoint	Results	Findings
**SAFER (2002) [18, 19] **	801	GuardWire vs. PCI	57.1% vs 58.7%	MACE (30 days)	9.6% vs 16.5% p=0.004	Embolic protection showed a 6.9% absolute risk reduction and a 42% relative risk reduction in the primary endpoint at 30 days. Not powered to show a significant reduction in mortality, but had a trend towards less mortality with embolic protection (1.0 vs 2.3% p=0.17).
**FIRE (2003) [20] **	651	FilterWire vs Guardwire	51.5 vs 53.3%	MACE (30 days)	9.9% vs 11.6% p=0.0008(NI)	No difference between the groups in the primary endpoint. FilterWire EX system shown to be non-inferior to Guardwire in percutaneous intervention of SVG.
**PRIDE (2005) [21] **	631	Triactive System vs Filterwire EX/GuardWire	54.0% vs 54.7%	MACE (30 days)	11.2% vs 10.1% p=0.02(NI)	No difference between the groups in the primary endpoint. Triactive system shown to be non-inferior to approved Guardwire and FilterWire devices in percutaneous intervention of SVG.
**AMEthyst (2008) [22] **	797	Interceptor Plus vs. GuardWire/FilterWire EZ	40% vs 39.4%	MACE (30 days)	8% vs 7.3% p=0.023 (NI)	No difference between the groups in the primary endpoint. Interceptor Plus embolic protection device shown to be non-inferior to approved Guardwire and FilterWire in percutaneous intervention of SVG.
**PROXIMAL (2007) [23] **	594	Proxis System vs GuardWire/FilterWire	42.5% vs 44.3%	MACE (30 days)	9.2% vs 10.0% p=0.0061(NI)	No difference between the groups in the primary endpoint. Proxis Embolic Protection System system shown to be non-inferior to distal embolic protection devices (GuardWire, FilterWire EX, and FilterWire EZ) in percutaneous intervention of SVG.

GP indicates Glycoprotein; MACE, Major Adverse Cardiac Events; NI, non-inferior; SVG, saphenous vein graft.

**Table 2.** Randomized Trials of EPD Usage in Native Vessel PCI for STEMI

Trial/Reference	n	Device	GP IIb/IIIa Use	Primary End Point	Results (Embolic protection vs PCI)	Findings
**EMERALD (2005) [32] **	501	GuardWire plus	83%	STR>70% 99mTc-Sestamibi Infarct Size	63.3% vs 61.9% p=0.78 12.0% vs 9.5% p=0.15	No significant difference in STR and infarct size. No difference in MACE between the two groups at 6 months.
**MICADO (2007) [38] **	154	Guardwire	Unknown	TIMI Perfusion Grade 3	58% vs. 44% p=0.054	Embolic protection group tended to show a better TIMI perfusion grade. No differences in MACE at 6 months between the groups.
**ASPARAGUS (2007) [39] **	341	GuardWire Plus	Unknown	STR>70% (90 minutes) MBG at 30 days	38.2% vs 35.5% p=0.81 42.9% vs 30.4% p=0.035	Less distal embolization with embolic protection but no significant difference in markers of myocardial damage. No difference in MACE at 30 days
**PREMIAR (2007) [40] **	140	SpideRX	26%	STR≥70%	61.2% vs 60.3% p=0.85	No statistically significant difference in myocardial reperfusion by angiography or ECG. No statistically significant difference in EF by echocardiography. No difference in MACE at 6 months.
PROMISE (2005)* [33]	200	FilterWire-EX	100%	Adenosine-induced flow velocity in IRA	34(± 17cm/s) vs 36(± 20cm/s) p=0.46	No improvement in perfusion by flow velocity and difference in infarct size between the two groups. No difference in MACE at 30 days.
**UPFLOW (2007) [41] **	100	FilterWire-EX	75%	TIMI 3 flow STR≥70% MBG 3	88.2% vs 93.9% p=NS 65.6% vs 64.3% p=NS 68.1% vs 66% p=NS	No difference in myocardial reperfusion by angiography or ECG. No difference in MACE at 30 day follow up.
**DEDICATION (2008) [34] **	626	FilterWire-EZ & SpiderX	96.33%	STR≥70% (90 minutes)	76% vs 72% p=0.29	No difference in myocardial reperfusion by ECG. No difference of MACE at 30 days.
**PREPARE (2009) [35] **	284	Proxis Embolic Protection System	39.10%	STR≥70% (60 minutes)	80% vs 72% p=0.14	More rapid STR in treatment group. No difference in myocardial reperfusion by MBG nor ECG at 60 minutes. MACE occurred with similar frequency in both groups.

* Only trial to include NSTEMI patients in addition to STEMI

Table **2**: 100% in control group (PCI); GP indicates glycoprotein; STR, indicates ST resolution; MBG, myocardial blush grade; TIMI, thrombolysis in myocardial infarction; PCI, percutaneous coronary intervention; MACE, major adverse cardiac events; LV, left ventricle; EF, ejection fraction; ECG, electrocardiogram; NSTEMI-non ST elevation Myocardial Infarction; STEMI, ST elevation myocardial infarction.
